# Case of William Freeman, the Murderer of the Van Nest Family

**Published:** 1847-11

**Authors:** 


*Case of William Freeman, the Murderer of the Van Nest Family.* By BLANCHARD FOSGATE, M. D., of Auburn, N. Y.

William Freeman—the murderer of the Van Nest family—was a native of Auburn, Cayuga Co., N. Y., twenty-three years old. In stature he measured about five feet seven inches, and when in health weighed in the vicinity of one hundred and fifteen pounds. He had a broad chest, and was of muscular make. With the exception of a slight admixture of aboriginal blood, he was of African descent.

At the age of sixteen he was sentenced to five years' imprisonment in the State prison at Auburn, for grand larceny. It was long since conceded that of this charge he was innocent. His sentence expired in September, 1845. He left his prison conscious of the injustice he had suffered, and had imbibed an idea that he was entitled to pay for his time. This sentiment could not be eradicated from his mind, and on several occasions he applied for warrants against those he supposed liable. Remuneration with him was the *one idea.* Failing in this mode of obtaining redress, he armed himself with a common butcher's knife, and a cane with a blade attached to the lower end, and from his lodging made his way toward the Owasco Lake, at about sunset on the 12th of March, 1846. After examining two or three premises, he finally selected the residence of Mr. Van Nest as the proper place to begin "his work," as he termed it, and there massacred Mr. Van Nest, his wife and one child, aged two years, and Mrs. Wyckoff, aged 70. He stabbed Mr. Vanarsdale in the chest, who subsequently recovered. In the affray he entered every room in the house, both above and below, but took nothing away. He went to the stable, unfastened and mounted a horse, and was some rods from the scene of devastation in the incredibly short period of not more than five minutes from the time of entering the house, as was proved in evidence. Three days afterwards he was committed to Cayuga county jail to await his trial.

He was tried at a special session of Oyer and Terminer, July, 1846—first, as to whether he was sane at the time of trial, and secondly, on the indictment. A verdict of *sufficient* soundness of mind to be put on trial was rendered on the preliminary issue, and of wilful murder on the indictment. Subsequently, however, a new trial was granted by the Supreme Court. A trial calling forth so much tallent in its prosecution, and arousing such fearful excitement among the people, is of rare occurrence.

On the part of the people, the cause was conducted by Hon. John Van Buren, attorney-general of the State of New York, and for the defence by Hon. William H. Seward; ex-governer of this State.

My knowledge of the prisoner commenced on the 16th of March 1845, being the day after his commitment, and it continued until the completion of a post-mortem examination of his body on the twenty-first of August, 1847.

During the scene at Van Nest's, he received a severe wound in the articulation of the right thumb with the carpus—the artery barely escaping division. This circumstance saved the lives of other members of the family, because, to use his own expression "he could'nt handle his hand any longer."

My services were required on account of this injury. In addition to the wound, I also found him entirely deaf in the left, and partially so in the right ear.

It was a singular circumstance that he never made an inquiry as to either the extent or condition of the injury, or the time necessary to complete a cure, or the prospect of recovering the use of his hand—though it was the right, and as a laborer was his main dependence. Neither did he complain of any sensibility in the wound, although the physical evidences of pain occompanying the inflammatory stage were such as to leave uo doubt of its existence. In fact from the time of his commitment until the day of his death, although he often saw, and was attended by me through his last sickness, he asked only two questions, one about his medicine, the other regarding his diet, and these were made during his last illness.

During the principal part of his incarceration, he passed his time standing; his body erect—his head a little drooping, and with arms folded. He sustained this posture with statue-like stillness—indicating great muscular strength. He exhibited a calm, quiet expression of countenance, occasionally broken by a smile, which had the appearance of just bursting into laughter, but would quickly subside leaving the same unalterable expression, as undisturbed as though a gleam of mirthfulness had never occupied his fancies. To the careless observer, it appeared as though he endeavored to suppress an irresistible propensity to laugh. This smile was never accompanied by any vocal sound, but often glowed upon his feature, regardless of time, place or circumstance, indicative of intense mental emotion. For this emotion he could never assign a cause. I say he never could, because, when asked, he always said he 'didn't know.' My conclusion is also based upon the remarkable fact, that on the trial *seventy two* witnesses on both sides coincided in the opinion, that the prisoner did not intend to deceive in any reply made to the numerous interrogatories put to him.

His deafness increased until the sense of hearing was nearly, if not quite, obliterated. I doubt whether he heard any conversation for the last two weeks of his life; at all events, I could not get a reply that harmonized with my question.

On the twelfth of April, 1847, I was called to see the patient as being "not very well." He had a quick thready pulse—considerable cough, with free expectoration—not much appetite, but rather thirsty. He made no allusion to these symptoms, but directed my attention to his left ear, which discharged pus profusely. From this time forth, the aural discharge continued, accompanied by all the symptoms of tubercular phthisis, until his existence terminated, six days after the chain that bound him to the masonry of his cell had been removed.

About three weeks previous to his decease, I observed a prominent protrusion of the left eye, and upon further examination there proved to be an entire obliteration of vision. He could not close the lids over it, for they, with all the muscles of that side of the face, were paralyzed, and the mouth considerably drawn to the right. The cornea of both organs had much the same appearance. The loss of vision, I am inclined to think, was the result of functional, not organic lesion. The protrusion depended most probably upon the loss of muscular power in its motor apparatus, in common with the muscles of that side of the face. The globe, in *articulo-mortis*, recovered in a great measure its natural location, as did the paralyzed muscles of the face—a common occurrence of facial distortion from nervous lesion at death.

Owing to insufficiency of light in the cell, but more particularly to the shattered condition of the patient—being deaf, almost blind, and nearly speechless—no satisfactory account of symptoms or the effect of remedies could be obtained from him.

As this case presents points of interest in many particulars, I would remark that phrenologically, Mr. Fowler says, "he is very defective in the mental temperament, and has great predominance in the muscular. His propensities (with the exception of self-esteem and firmness, very large—and combativeness and destructiveness, large) are all small, and have but little influence. The intellectual faculties are not so small, yet the quality of brain considered, their influance is quite limited. He has one of the most imperfect developments of brain I ever saw. He has no real balance to his mind; it is entirely onesided, he being at the mercy of circumstances, and the stronger propensities." (See *Phrenological Almanac* in press for 1848.) Another phrenologist, though of less notoriety, has allowed him a much better development; but whatever the external evidences of mind the contour of his head may denote, they all have reference to a healthy brain.

I have measured his cranium in two ways: First, by passing a string across the frontal and around the spinious process of the occipital bones. It measured, in the greatest circumference, twenty one inches. Secondly, after the directions laid down in Combe's phrenology by Callipers.

| | | |
|---|---|---|
| Viz. from occipital spine to individuality | $7\frac{3}{8}$ | inches |
| " occipital spine to ear | 4 4-8 | " |
| " ear to individuality | 4 6-8 | " |
| " ear to firmness | 5 3-16 | " |

" destructiveness to destructiveness $5\frac{3}{8}$ inches

" cautiousness to cautiousness 4 7-16 "

" ideality to ideality $5\frac{1}{8}$ "

On proceeding to a post-mortem examination, the body was found extremely emaciated. The costal and pulmonary pleura, though easily separated, were extensively adhered, and the lungs were an almost entire mass of disease. Tuberculous matter was interspersed with abscesses throughout the whole organ. The pericardium contained about one and a half gills of serum. The heart contained polypi, but had a healthy appearance. Liver natural. Gall bladder a little distended. Mucous membrane of the stomach slightly inflamed. Intestinal mucous coat healthy. Mesenteric glands turberculous. Urinary bladder distended. Kidneys natural. The peritoneum appeared healthy, but the sac contained some fluid.

Upon opening the cranium, the bones were found rather thinner than ordinary, particularly for a colored subject, and the dura mater was adherent to a portion of the occiput. The anterior portion of this membrane was congested and inflamed, with considerable serum between it and the arachnoid. This latter tunic was somewhat thickened and congested. The anfractuosities of the right hemisphere of the cerebrum were filled with serum. The superficial vessels of the right anterior lobe highly congested on the superior surface. Cerebellum to all appearance healthy.

The whole brain, separate from the dura mater, weighed $43\frac{3}{4}$ ounces avoirdupois. Cerebrum 38 ounces. Cerebellum $5\frac{3}{4}$ ounces.

On section of the medullary substance, it was found thickly stud ded with bright red points. The right thalami appeared to have undergone some change, and the whole superior brain was more or less congested. The membrane covering the petrous portion of the left cavity was congested, and the remaining parts of it appeared healthy.

There was caries of the inner part of the petrous portion of the left temporal bone. The membrana tympani, with the internal structure of the ear, most obliterated. There was a necrosis containing fetid pus, having no perceptible connection with the external ear.

*Remarks.*—The important question connected with this subject is, whether the pathological state of the brain, its membrances and the ear, was one of long standing or of recent occurrence? On this point rests the physical evidence of the prisoner's accountability.—If by possibility it could be determined that the organ of mental manifestation was without disease when the crime was perpetrated, then depravity unparalleled must be assigned as the only cause; and if so, the disease of the organ at his decease could not be held in extenuation of his crimes.

That the diseased condition of the brain was of long standing, appears to be unquestionable from the fact, that the mental organ could not sustain so great a lesion as the autopsy presented, without the mind having exhibited sudden and violent derangement, as well as other symptoms which accompany its acute diseases.—This, however, was not the case. He never complained of, or exhibited the ordinary symptoms in such instances, nor even gave evidence of any mental change whatever; but on the contrary, presented the same characteristics throughout. During his last sickness, there was not a single symptom indicating acute inflammation of the brain, and yet, on examination after death, there were abundant and unequivocal evidences of inflammatory action there.

The disease of the ear also was chronic, and dated its commencement some months previous to the commission of the crime. On his trial it was proved in evidence that about two years previous—when an inmate of the state prison—he was struck on the head with a board, the blow splitting the weapon into fragments. He attributed his deafness to this cause, or, to give his own description, "it knocked his words down his throat—his ears dropped down—his kernels (meaning the tonsils) dropped." Now the infliction of this blow upon a thin skull, associated with his own account of its effects, would lead us to conclude that the concussion seriously injured the auditory apparatus. It possibly burst the tympanum, and if so, it opened a communication between the external ear and the fauces, which induced the remark that "it knocked his words down his throat," &c. Is it not a just conclusion, that from this injury the diseased action was set up which ultimately involved the whole brain?

Whether the facial paralysis was the result of cerebral congestion, or whether it was owing to a diseased state of the nerves of motion in connection with the condition of the ossa petrosa, may be questionable, because the nerves, as they passed off the brain, were apparently healthy; but the right hemisphere of the brain being the most deeply implicated in the organic derangement, the paralysis would appear, as it did in this case, in the muscles of the opposite side.

It should not be forgotten, that the deceased had passed through scenes of blood seldom equaled, where but a single individual was the aggressor; that he had been surrounded by the wild fury of an enraged populace for hours; that he had been chained, and for a portion of the time bedded upon the stone floor of a dimly-lighted cell, for almost eighteen months; suffering the jeers and grimaces of inhuman and uncounted spectators; wasting by a slow process of consumption; sustaining the blight of one physical energy after another; with little compassion and less than ordinary attention; and through the whole period, having scarcely asked a question regarding either friend or foe, soliciting no favor, showing no hatred, exhibiting no remorse, entering no complaint, and through all, sustaining an *undisturbed tranquility.*

From this concatenation of circumstances, this unruffled, equable, almost idiotic state of mind, that no external relation could disturb, or internal influence alter, we can scarcely come to any other conclusion by pathological reasoning, than that the state of mind which he exhibited subsequent to his arrest, depended on a chronic derangement of the mental organ, and must have existed antecedent to the crime itself. If such a combination of pathological facts, and all the other circumstances attending the prisoner from his arrest to his death, do not establish an unsound state of mind, they at least present one of the most extraordinary cases furnished by the annals of our race. Such a casè demands the careful consideration of the philosopher and jurist.

How much the cause of justice and philosophy is indebted to the unwearied perseverance of the eminent advocate who withstood the tide of popular indignation in conducting the prisoner's defence, is left for other hands to register; but true it is, that over prejudice and ignorance, science has gloriously triumphed.

*Auburn, N. Y., Sept.* 1, 1847. [*Am. Jour. Med. Science.*

